# Are activated B cells involved in the process of myocardial fibrosis after acute myocardial infarction? An in vivo experiment

**DOI:** 10.1186/s12872-020-01775-9

**Published:** 2021-01-06

**Authors:** Fanrui Mo, Ying Luo, Yuluan Yan, Juan Li, Shayi Lai, Weifeng Wu

**Affiliations:** 1 Department of Cardiology, The First Affiliated Hospital of Guangxi Medical University, 6 Shuangyong Road, Nanning, 530021 China; 2 Department of Cardiology, Fourth Affiliated Hospital of Guangxi Medical University, Liuzhou, China; 3 Guangxi Medical University, Nanning, China

**Keywords:** Acute myocardial infarction, Activated B cells, Cytokines, Collagen, Left ventricular function

## Abstract

**Background:**

Inflammatory cells infiltrate into the ischemic and hypoxic myocardial tissue after myocardial infarction. B cells gather at the site of myocardial injury and secrete cytokines to regulate immune inflammation and fiber repair processes.

**Methods:**

The animal experiment used ligation of the left anterior descending (LAD) artery of C57BL/6 mice to establish a mouse acute myocardial infarction (AMI) model to observe changes in activated B cells and cytokines at different time points. Twelve-week-old C57BL/6 male mice were randomly divided into the Sham group (24 mice) (thread under the LAD artery without ligation) and the AMI group (64 mice). In addition, C57BL/6 B-cell knockout (BKO) mice and C57BL/6 wild-type (WT) mice were used to establish AMI models to observe the expression levels of cardiomyocyte cytokines, such as TNF-α IL-1β, IL-6, TGF-β1, COL1-A1, COL3-AIII, TIMP, and MMP9. Moreover, pathological and collagen changes in the myocardium were analysed. One-way ANOVA and LSD method was used for comparisons of multiple and pairwise groups respectively. *P* < 0.05 indicated significant differences.

**Results:**

An AMI model of C57BL/6 mice was established successfully. The ratio of activated B cells and the expression of TNF-α, IL-1β, IL-6, TGF-β1, and B cell activating factor (BAFF) in the 5-day subgroup were the highest in the myocardium, spleen and peripheral blood with the most obvious myocardial inflammatory cell infiltration. The cytokines mRNA expression levels in the 5-day subgroup of the BKO group were decreased compared with those in the WT group (*P* < 0.05). Among the 2-week subgroups of the Sham, WT and BKO groups, the the LVEDd and LVESd of the BKO group were lower than those of the WT group (*P* < 0.05), and the left ventricular ejection fraction was higher than that of the WT group (*P* < 0.05).

**Conclusion:**

Activated B cells participate in the sustained state of myocardial inflammation and immune system activation after AMI, and may affect the metabolism of myocardial collagen after AMI by secreting cytokines. Moreover, B cells promote the expression of myocardial collagen Type I and Type III and damage the left ventricular ejection function.

## Background

Acute myocardial infarction (AMI) can lead to myocardial fibrosis after myocardial injury and morphological changes, such as impaired left ventricular function, left ventricular remodelling and heart enlargement [1]. Although the success rate of AMI treatment is increasing with the widespread implementation of emergency percutaneous coronary intervention (PCI) and thrombolytic therapy, 40% of patients have left ventricular remodelling, and 14.2% have heart failure [2]. Studies have found that AMI vascular recanalization still cannot prevent the progression of myocardial fibrosis in some patients after recanalization [3]. Heart failure and arrhythmias caused by myocardial fibrosis seriously threaten human life and health. Once pathological remodelling occurs, it is difficult to reverse. There is currently a lack of effective treatment methods, and new treatment methods are urgently needed to solve this problem.

With the rise of precision medicine, immune-related treatments such as molecular targeted drugs have become a research hotspot. Increasing evidence suggests that activation of myocardial inflammation and the immune system after AMI exacerbates the process of myocardial fibrosis [4]. After myocardial infarction (MI), a large number of inflammatory cells infiltrate the ischaemic and hypoxic myocardial tissue. Neutrophils and monocytes-macrophages are responsible for cleaning the necrotic tissue and secreting pro-inflammatory cytokines, such as TNF-α, IL-1β, and IL-6, and anti-inflammatory cytokines, such as TGF-β1. Then, T cells and B cells gather at the site of myocardial injury and secrete cytokines to regulate immune inflammation and fibrosis repair processes. Although inflammation and fibrosis are the basic physiological responses for healing and repair after tissue injury, excessive inflammation and fibrosis lead to left ventricular remodelling after MI [5, 6]. The involvement of innate immunity in tissue fibrosis has been recognized by the public [7], and there have been many studies on the involvement of T cells in myocardial fibrosis in adaptive immunity. However, as another important member of the adaptive immune system, the role of B cells in myocardial injury and fibrosis has only been gradually studied in recent years. B cells are an important type of adaptive immune cells that are mainly involved in humoral immunity and have the regulatory effects of producing antibodies, presenting antigens and secreting cytokines [8]. Recent studies have found that B cells are involved in the process of cardiovascular disease through an extensive immune regulatory network. Nish-Imura et al. [9] proved in a mouse model of dilated cardiomyopathy with PD-1 deficiency that PD-1, an important factor related to B-cell-specific differentiation, was deficient, and severe spontaneous dilated cardiomyopathy occurred in mice. In addition, activated B cells can cause myocardial damage through regulated death signaling pathways and complement-mediated cytotoxicity [10]. Most of these studies focus on B cells secreting antibodies to participate in MI and myocardial injury, but the role of secreting cytokines, another important function of B cells, in this process is not well studied. The mechanism is not clear, especially in the study of the immune response and fibrosis progression after AMI.

Previous studies have confirmed that B cells can promote the secretion of TGF-β1, TNF-α, IL-6, and other inflammatory factors [8, 11–16]. These factors are involved in myocardial injury and repair. Related studies have found that inflammatory cytokines play an important role in promoting or inhibiting tissue fibrosis in the process of the fibrosis cascade reaction. TGF-β1 is a pro-inflammatory factor in major tissues and organs that can phosphorylate Smad2/3 protein reactants, stimulate innate immune cells and activate fibroblasts to produce more cytokines to promote the occurrence and development of tissue fibrosis [17]. In addition, IL-6 and TNF-a can also act indirectly through TGF-β1 inflammation and tissue fibrosis pathways [18]. In an AMI mouse model, NLRP3 inflammasomes can activate the formation of IL-1β, cause myocardial damage and myocardial fibrosis, and directly inhibit the formation of NLRP3 inflammasomes. The results show that the area of MI is reduced and left ventricular function is improved [19]. In summary, B cells participate in the local inflammatory state of MI after AMI and secrete cytokines to participate in it, among which pro-inflammatory factors can promote fibrosis.

In recent years, it has been confirmed that B cells are involved in the process of tissue fibrosis in studies on transplantation immunity [20], autoimmune diseases [21], liver fibrosis [22], pulmonary fibrosis [23] and other diseases. In autoimmune diseases, the use of anti-BAFF and anti-CD20 antibodies can induce the depletion of pre-B cells, which can reduce the degree of tissue fibrosis [24]. In non-cardiovascular diseases, anti-B-cell therapy can reduce fibrosis, which gives us a new idea as to whether anti-B-cell therapy can also reduce fibrosis in AMI. To answer this question, we first need to explore the effects of B cells on AMI myocardial fibrosis. Studies have found that in mouse AMI models, there is a dynamic evolution of B-cell infiltration in the injured local myocardial tissue [25].

Therefore, we propose that activated B cells participate in the process of myocardial fibrosis after AMI. We intended to study this subject through animal experiments to determine the specific mechanism of myocardial fibrosis caused by activation of myocardial fibroblasts, which might provide potential new targets in immunotherapy for the prevention and treatment of myocardial fibrosis caused by AMI.

## Methods

### Animal sample size and randomization

Twelve-week-old C57BL/6 male wild-type (WT) mice were provided by the Experimental Animal Center of Guangxi Medical University. B-cell knockout (BKO) mice were purchased from Jackson Laboratory, USA, and the animals were kept in the SFP animal room of the centre. Animal experiments followed the Guidelines for the Use of Experimental Animals formulated by the National Institutes of Health of the United States. The research plan was approved by the Experimental Animal Ethics Committee of The First Affiliated Hospital of Guangxi Medical University.

 A total of 88 12-week-old C57BL/6 male mice (20–25 g) were randomly divided into the Sham group (24 Sham mice) and the AMI group (64 AMI mice). The two groups were divided into 4 subgroups at time points of 3 days, 5 days, 1 week and 2 weeks. There were 6 mice in each subgroup of the Sham group and 12 mice in each subgroup of the AMI group to ensure that each AMI subgroup ended with at least 8 mice alive.

 The 30 WT C57BL/6 mice were divided into the Sham group (12 Sham mice) and AMI group (18 WT C57BL/6 AMI mice). Eighteen BKO mice were included in the BKO group. The three groups were divided into two subgroups: a 5-day group and a 2-week group.

### Models and treatment

We injected 1.25% avertin into the abdominal cavity of the mice under general anaesthesia at a dose of 0.2 ml/10 g. After the mice were breathing smoothly, the skin on the left chest was incised, the pectoralis major and pectoralis minor muscles were bluntly separated, and the thorax was exposed. Then, the thorax was opened between the side 3–4 intercostals to expose the heart with a mouse chest opener. Size 8-0 nondestructive sutures were used to sew from right to left on the anchor point 2 mm from the lower edge of the left atrial appendage. The depth of the needle was approximately 1 mm, and the width was approximately 1–1.5 mm. Next, the needle holder was knotted, and the left anterior descending (LAD) artery was permanently ligated. After the ligation, the myocardium became white, and the movement was weakened at the bottom of the suture. A 1-ml syringe is pumped back into the thoracic cavity to develop negative pressure. Finally, the muscles and skin of the chest and neck were sutured layer by layer using 5-0 sutures. When the mice regained spontaneous breathing, tracheal intubation was removed, and they were kept warm and returned to the cage for rearing. The mice in the Sham group were subjected to conventional open-chest surgery. The hearts were left open and threaded under the LAD artery without ligating the blood vessels. The rest of the operation was the same as previously described.

### Preparation of peripheral blood monocyte suspension

Peripheral blood (1.5 ml) from mice was collected into pretreated heparin anticoagulant EP tubes by drawing blood from the eyeball.

After 20 min of standing at room temperature, the peripheral blood was stratified. The upper yellow serum was collected into the EP tube and stored in a − 80 °C refrigerator. The lower layer of blood cells was transferred into a 5-ml BD flow tube, and then 2 ml of diluted red blood cell lysate was added. The mixture was blown and mixed until the mixture was mixed, and the mixture was allowed to stand for 5 min at room temperature. After centrifugation of the mixed mixture at 300×*g* for 5 min, 2 ml of sterile PBS was added to stop lysis. Then, the supernatant was discarded after centrifugation at 300×*g* for 5 min once again. The cells were washed with PBS and centrifuged for the third time, and the supernatant was discarded. Then, 1 ml of PBS was added to the resuspended cells. If the cell count was up to standard, the sample was set aside as a reserve.

### Preparation of a spleen single-cell suspension

Mice were sacrificed by neck dislocation. The spleen was removed aseptically and transplanted into a 1.5-ml aseptic EP tube, followed by the addition of 300 μl of aseptic PBS into the EP tube. The spleen was gently ground with a sterile glass rod until it was white and transparent. The ground tissue solution was filtered through a 200-μm filter screen, and the filtered spleen tissue cell solution was collected and transferred into a sterile test tube. After centrifugation of the spleen tissue cell solution at 300×*g* for 5 min, the supernatant was discarded. Next, 1 ml of diluted red blood cell lysate was added to it and then blown and mixed until the cells mixed well, after which the mixture was incubated for 5 min at room temperature. Then, the samples were centrifuged at 300×*g* for 5 min, and 1 ml of sterile PBS was added to stop lysis. Then, the cells were centrifuged at 300×*g* for 5 min once again, the supernatant was discarded, and the cells were washed with PBS. Then, the supernatant was centrifuged two more times, and 1 ml of PBS was added to resuspend the cells in reserve.

### Preparation of a myocardial single-cell suspension

After the heart was removed under aseptic conditions, it was rinsed with PBS buffer repeatedly. The blood inside the heart was gently squeezed until it was clean. Then, the heart was transferred into a 1.5-ml sterile EP tube with 300 μl of sterile PBS. The ventricular tissue was cut into pieces with ophthalmic scissors and subsequently placed into a 15-ml centrifuge tube. Next, 3.0 ml of collagenase IV was added, and the tube was placed into a shaking table for digestion at 37 °C for 30 min. Myocardial tissue was repeatedly blown with a 3.0-ml straw to fully mix the tissue with digestive enzymes. After filtration with a 100-mesh aperture nylon mesh, the cells were collected and added into BD flow tubes. After centrifugation of the tissue solution at 1500 rpm/min for 5 min, 1 ml of diluted red blood cell lysate was added to resuspend the cells. Then, the cells were incubated for 5 min at room temperature, and 1 ml of PBS was added to neutralize the cells.

Subsequently, the samples were centrifuged at 1500 rpm for 5 min once again, the supernatant was discarded, and the cells were washed with PBS and centrifuged twice. Finally, 1 ml of PBS was added to resuspend the cells. If the cell count was up to standard, the sample was set aside as a reserve.

### Flow cytometry

One hundred microlitres (containing approximately 1 × 10^6^ cells) of a single-cell suspension of peripheral blood and spleen and myocardial tissue was taken and placed in a centrifuge tube. Then, 1 μl of anti-MOUSE CD19-PERCp-CYANine5.5 antibody (BD Biosciences) and 1 μl of anti-mouse CD69 PE antibody (BD Biosciences) were added into the above 3 suspension tubes. The suspension was incubated at 4 °C for 30 min. Subsequently, we added 1 ml of PBS to the suspension and centrifuged it at 300×*g* for 5 min. The cells were resuspended in 300 μl of 4% paraformaldehyde after the supernatant was discarded. We used BD company FACS Canto II instruments to conduct flow cytometry. The lymphocytes were P1, and CD19 was P2. The expression ratio of activated B cells was determined according to the proportion of CD69 in B cells. Flow cytometry results were analysed with FlowJo 7.6.1 software. FlowJo is Copyright © Trustees of Leland Stanford Jr. University, 1996–2019.

### Real-time fluorescence quantitative polymerase chain reaction (RT-PCR)

Total RNA was extracted from myocardial tissue samples of AMI mice and control mice by using TRIzol™ reagent according to the manufacturer’s instructions (Bao Biological Engineering Co., Ltd.). RNA was dissolved in sterile water and quantified by NanoDrop2000 ultraviolet spectrophotometry at 260 nm/280 nm (A260/A280 in 1.9–2.1 was considered the qualified purity), after which it was reverse-transcribed using All-in-One cDNA Synthesis SuperMix. RT-PCR was performed using a Reverse Transcript Kit (Bao Bioengineering Co., LTD). The PCR conditions were 30 s at 95 °C followed by 40 cycles at 95 °C for 5 s, 58 °C for 30 s, and 72 °C for 30 s. The relative expression levels of genes were calculated by the ΔΔCt value. The primer sequences, including TGF-β1, TNF-α, IL-6, IL-1β, BAFF and β-actin, are presented in Table 1.

**Table 1:** The primer sequences applied in our study

Gene	Tm (°C)	Forward sequence	Reverse sequence
TGF-β1	62.5	ATGTCTCAGCCTCTTCTCATTC	TGGTGAATGACAGTGCGGTTATGG
TNF-α	58.2	ATGTCTCAGCCTCTTCTCATTC	GCTTGTCACTCGAATTTTGAGA
IL-6	60.5	CTCCCAACAGACCTGTCTATAC	CCATTGCACAACTCTTTTCTCA
IL-1β	60.5	TCGCAGCAGCACATCAACAAGAG	TGCTCATGTCCTCATCCTGGAAGG
BAFF	53.9	AACAAGATGTAGACCTCTCAGC	CTGCAGACAGTCTTGAATGATG
β-actin	60.5	CTACCTCATGAAGATCCTGACC	CACAGCTTCTCTTTGATGTCAC
COL1A1	54.3	AAAGATGGACTCAACGGTCTC	CATCGTGAGCCTTCTCTTGAG
COL3A1	52.4	GAAAGAATGGGGAGACTGGAC	TACCAGGTATGCCTTGTAATCC
TIMP-1	54.2	CTCCAGTTTGCAAGGGATAGAT	CAAAGACCTGAAAACCTCCAAC
MMP-9	53.9	CAAAGACCTGAAAACCTCCAAC	GACTGCTTCTCTCCCATCATC

### Detection of TNF-α, IL-1β, IL-6, BAFF and TGF-β1 in peripheral blood.

TNF-α, IL-1β, IL-6, BAFF and TGF-β1 concentrations were measured with enzyme-linked immunosorbent assay (Novus Biologicals) according to the instructions.

### Echocardiography of heart

Mice in each group were intraperitoneally injected with 1.25% Afertin general anaesthetic according to time points and laid in the supine position on an operating table for small animals. A parasternal minor axis section examination was performed. The left ventricular end diastolic dimension (LVEDd), left ventricular end-systolic dimension (LVESd) and left ventricular ejection fraction (LVEF) values of the mice were recorded with M-mode ultrasound.

### Statistical analysis

All data are presented as the mean ± standard deviation, one-way ANOVA was used for comparisons between groups, and the LSD method was used for pairwise comparisons between groups. *P* < 0.05 was considered statistically significant.

## Results

### Effect of activated B cells on myocardial fibrosis in mice with acute myocardial infarction

#### General situation of the mice

**Fig. 1:**
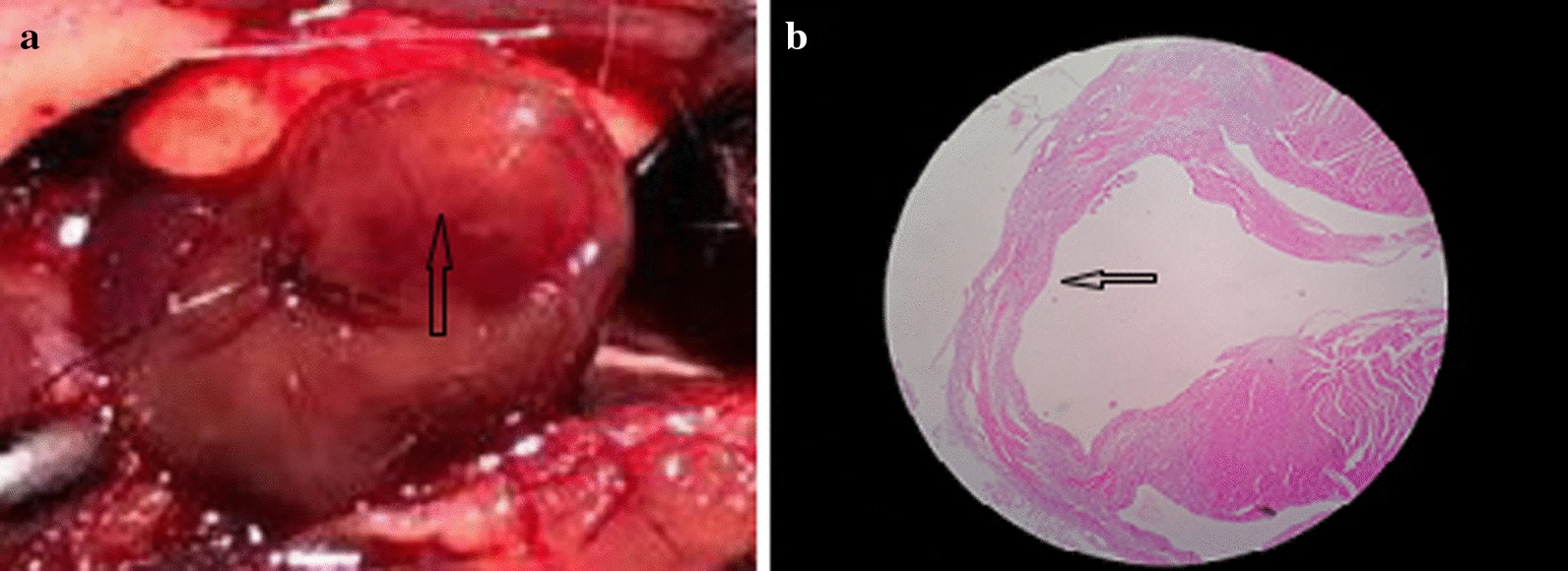
Gross specimen and pathological features after LAD ligation in mice. **a** Myocardial whitening below ligation line after ligation of LAD in mice (arrow indication), **b** HE staining after myocardial infarction suggested myocardium thinning (arrow indication). *LAD* left anterior descending, *HE* hematoxylin–eosin staining

After AMI, the mice were fully awake, the mice showed reduced activity and no obvious stimulus response, while the mice in the Sham group generally responded normally. Ten mice in the AMI group died, including 2 mice in the 3-day and 1-week subgroups and 3 mice in the 5-day and 2-week subgroups. No mice died in the Sham group. Gross specimen and pathological findings of mice after LAD artery ligation are shown in Fig. 1.

#### Ratio of activated B cells in mouse myocardium, spleen and peripheral blood

**Fig. 2:**
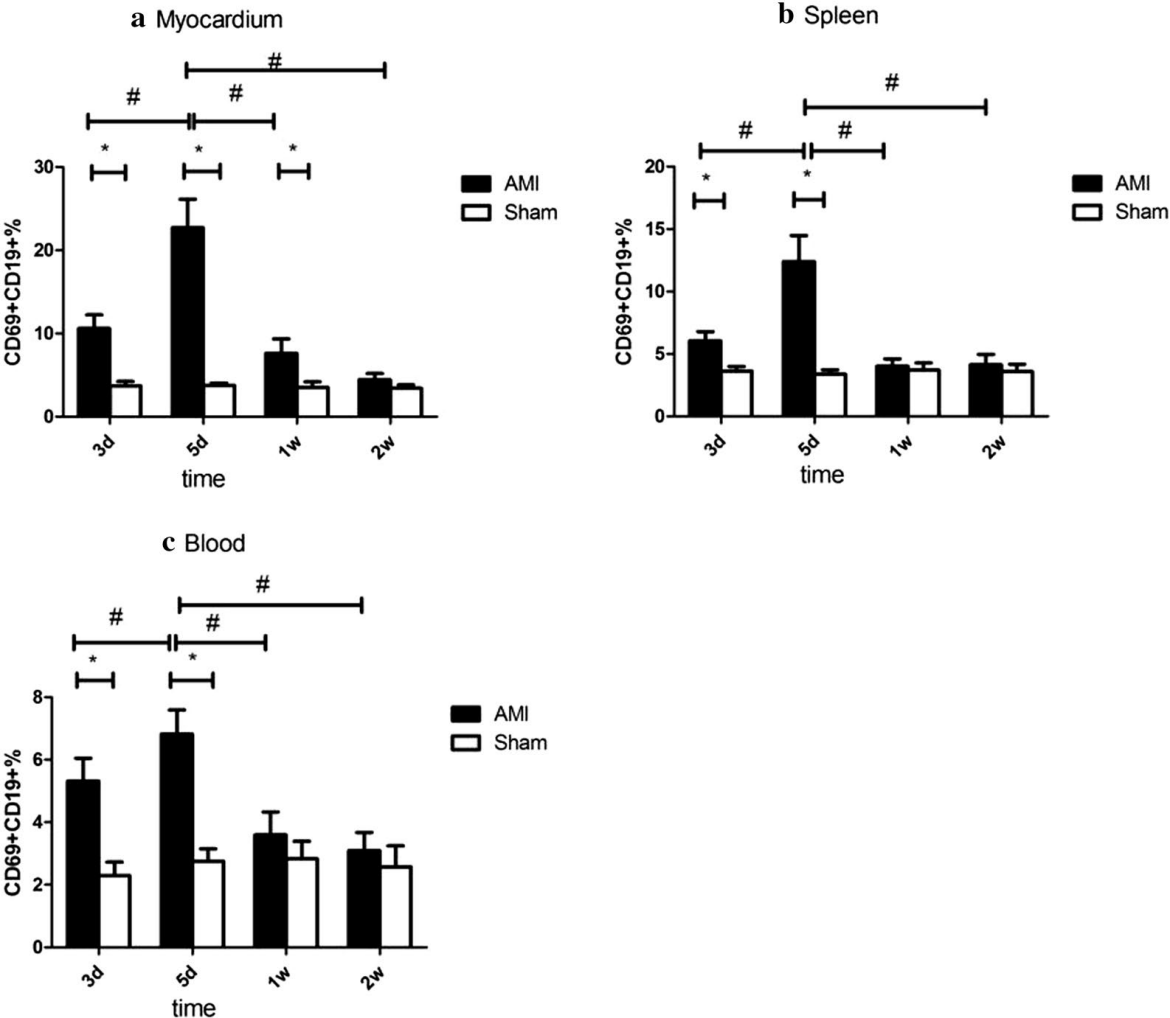
Results of activated B-cell flow in myocardium, spleen, and peripheral blood of the AMI group and Sham group in different subgroups. *P < 0.05, the activated B cell ratio of AMI group was compared with the corresponding subgroups of the Sham group, ^#^*P* < 0.05, the activated B cell ratio of other subgroups of the AMI group compared with AMI 5 days subgroups. Data are represented by mean ± standard deviation. *AMI* acute myocardial infarction

**Fig. 3:**
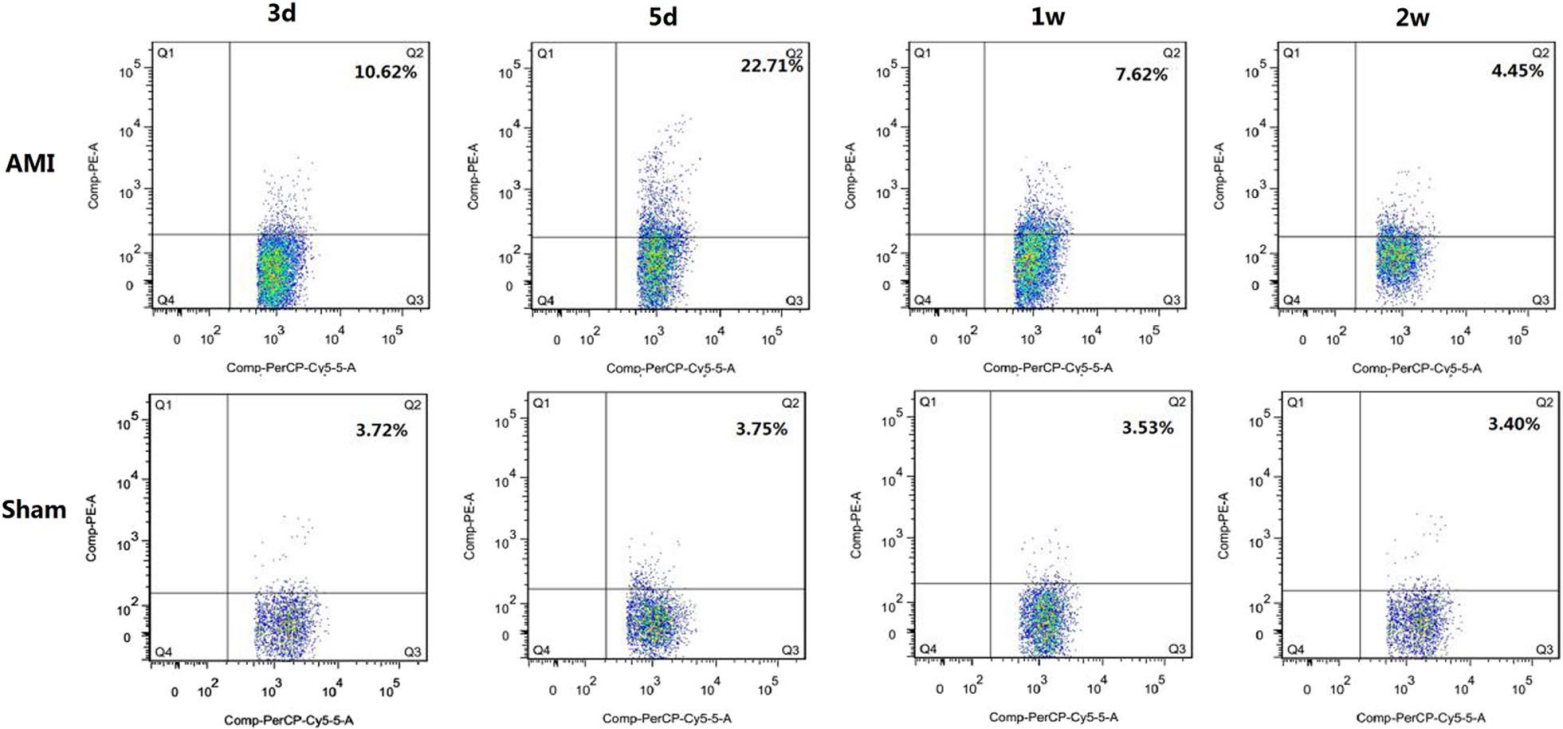
Flow cytometry of myocardial activation B cells in the AMI group and Sham group in different subgroups. The numbers in the upper right quadrant represent the mean proportion of activated B cells. *AMI* acute myocardial infarction

In myocardial tissue, the proportion of activated B cells in the AMI group was higher than that in the Sham group in the 3-day, 5-day, and 1-week subgroups (*P* < 0.05), there was no significant difference from the Sham group in the 2-week subgroup (*P* > 0.05), and the highest level was represented in the 5-day subgroup (*P* < 0.05). In the spleen and peripheral blood, the proportion of activated B cells in the AMI group was higher than that in the Sham group in the 3-day and 5-day subgroups (*P* < 0.05), and the highest level was represented in the 5-day subgroup (*P* < 0.05). There was no significant difference between the 1-week and 2-week subgroups and Sham groups (*P* > 0.05) (Table 2, Figs. 2, 3).

**Table 2:** The percentages of CD69^+^ CD19^+^ cells in myocardium, spleen, and blood in AMI and control mice

Group	n	CD19^+^ CD69^+^ (%)		
		Myocardium	Spleen	Blood
AMI 3d	8	10.62 ± 1.62*^#^	6.05 ± 0.73*^#^	5.32 ± 0.73*^#^
Sham 3d	6	3.72 ± 0.51	3.62 ± 0.36	2.29 ± 0.43
AMI 5d	8	22.71 ± 3.43*	12.40 ± 2.07*	6.82 ± 0.77*
Sham 5d	6	3.75 ± 0.29	3.80 ± 0.40	2.76 ± 0.39
AMI 1W	8	7.62 ± 1.74*^#^	4.02 ± 0.58*^#^	3.60 ± 0.73^#^
Sham 1W	6	3.53 ± 0.65	3.71 ± 0.56	2.84 ± 0.55
AMI 2W	8	4.45 ± 0.75^#^	4.13 ± 0.82^#^	3.10 ± 0.58^#^
Sham 2W	6	3.40 ± 0.44	3.58 ± 0.59	2.58 ± 0.67

^*^P < 0.05, the activated B cell ratio of AMI group was compared with the corresponding subgroups of the Sham group

^#^*P* < 0.05, the activated B cell ratio of other subgroups of the AMI group was compared with AMI 5 days subgroups. Data are represented by mean ± standard deviation

#### Expression of cytokine mRNA in cardiomyocytes of AMI mice

**Fig. 4:**
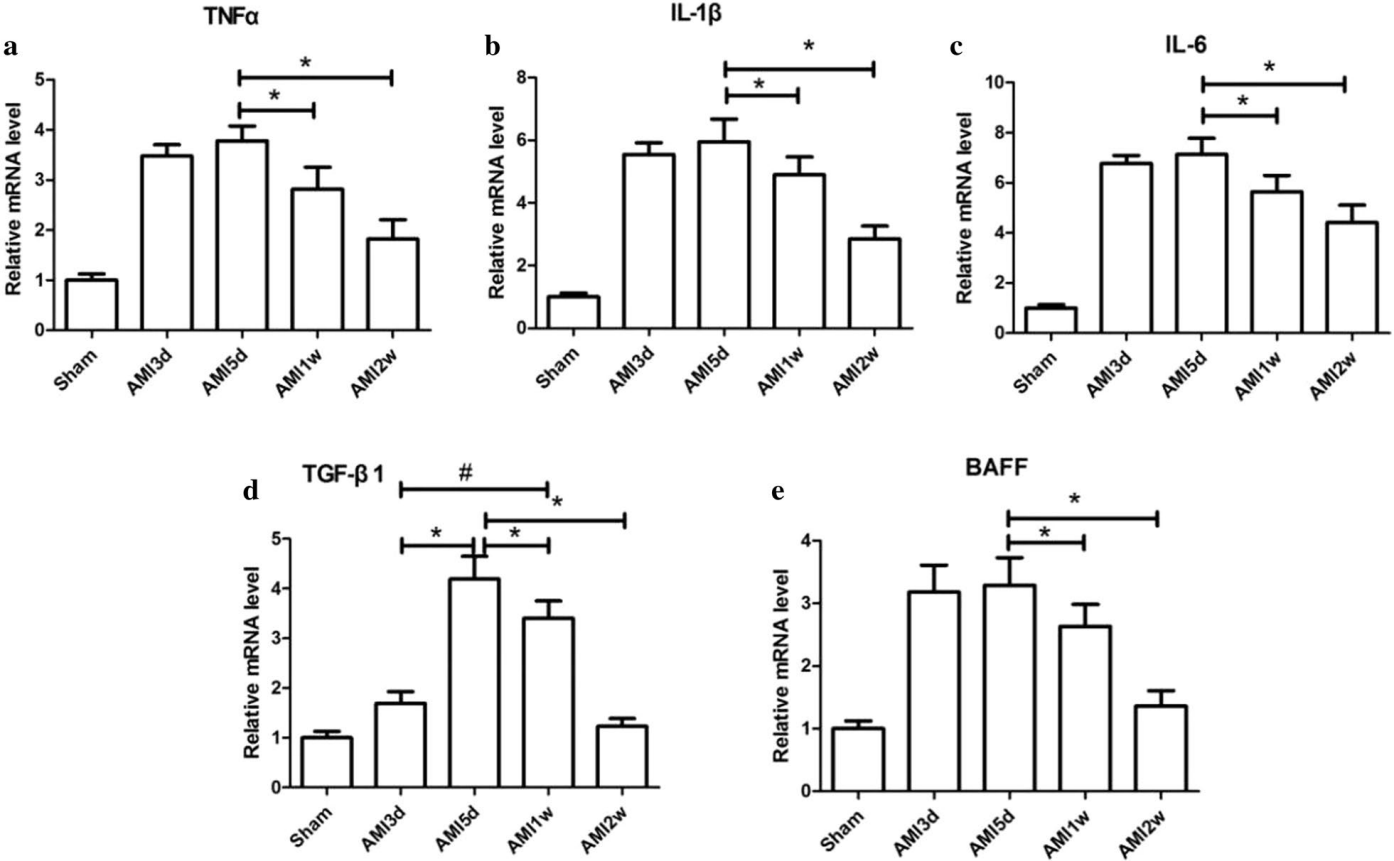
Relative expression levels of cytokine mRNA in AMI mice myocardium. *P < 0.05, the relative expression levels of cytokine mRNA of other subgroups was compared with AMI group 5-day subgroup. ^#^*P* < 0.05, the relative expression levels of cytokine mRNA of AMI 1-week subgroup was compared with AMI 3-day subgroup. *AMI* Acute myocardial infarction. *TNF-α* tumour necrosis factor-α, *IL-1β* interleukin-1β, *IL-6* interleukin-6, *TGF-β1* transforming growth factor-β1, *BAFF* B cell activating factor

In myocardial tissue, the mRNA levels of TNF-α, IL-1β**,** IL-6 and BAFF in the AMI group were higher than those in the Sham group at 3 days, 5 days, 1 week, and 2 weeks (*P* < 0.05) and were highest in the 3-day and 5-day subgroups (*P* < 0.05); however, the mRNA levels of TNF-α, IL-1β**,** IL-6 and BAFF in the AMI 3-day and 5-day subgroups were not statistically significant (*P* > 0.05). The level of TGF-β1 was higher than that in the 4 subgroups in the Sham group, and the highest level appeared in the 5-day subgroup (*P* < 0.05) (Fig. 4).

#### Levels of cytokines in the peripheral blood of AMI mice

**Fig. 5:**
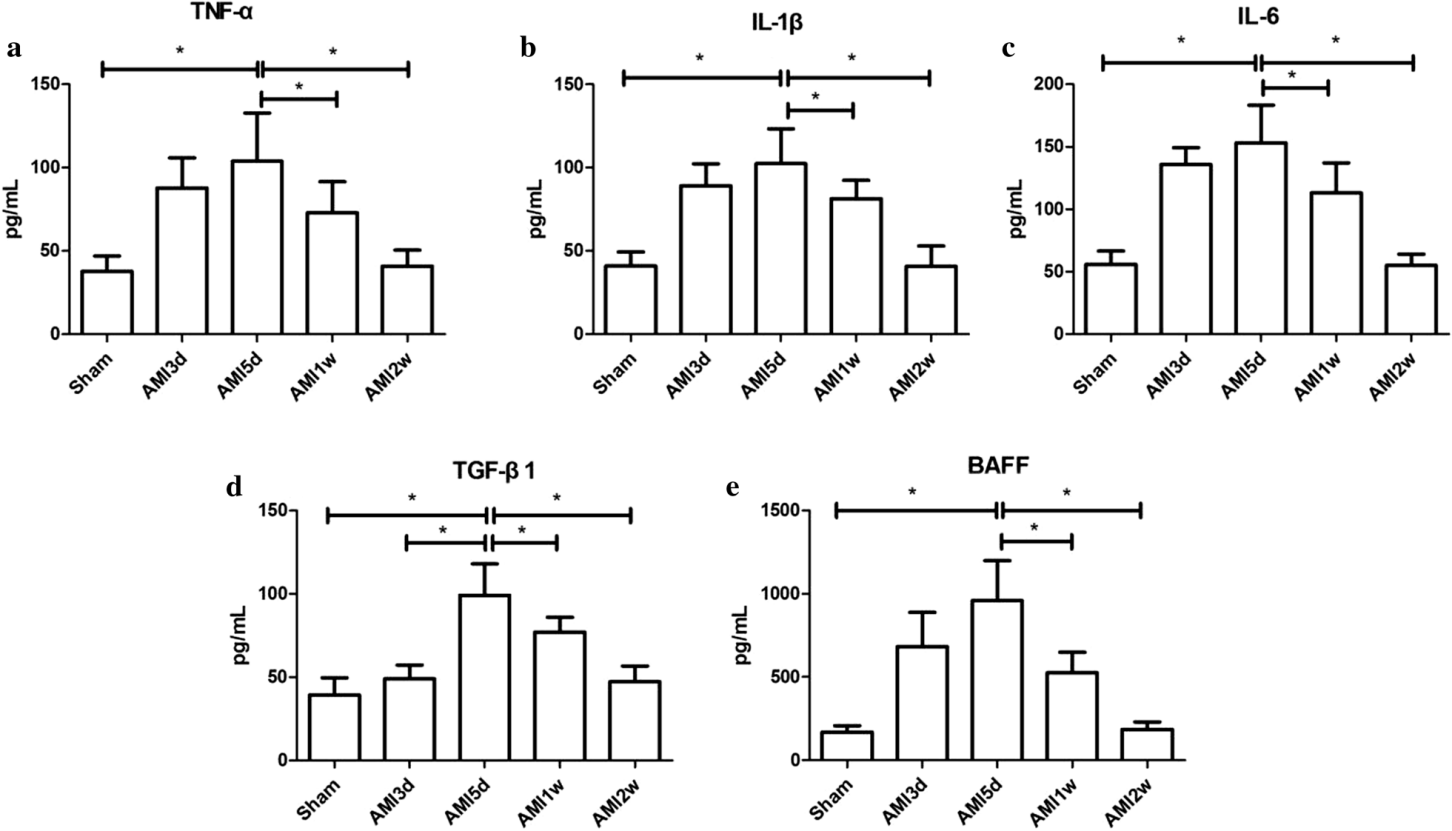
Peripheral blood cytokine concentration of AMI group. *P < 0.05, the peripheral blood cytokine concentration of other subgroups were compared with AMI 5-day subgroup. *AMI* acute myocardial infarction, *TNF-α* tumour necrosis factor-α, *IL-1β* interleukin-1β, *IL-6* interleukin-6, *TGF-β1* transforming growth factor-β1, *BAFF* B cell activating factor

The concentrations of TNF-α, IL-1β**,** IL-6 and BAFF in peripheral blood in the AMI group were higher than those in the Sham group, including the 3-day, 5-day, 1-week, and 2-week subgroups (*P* < 0.05), and were highest in the 3-day and 5-day subgroups (*P* < 0.05). TGF-β1 concentrations were higher in the 4 subgroups than in the Sham group, with the highest concentration in the 5-day subgroup (*P* < 0.05). The results are shown in Fig. 5.

#### Pathological changes in mouse myocardium

**Fig. 6:**
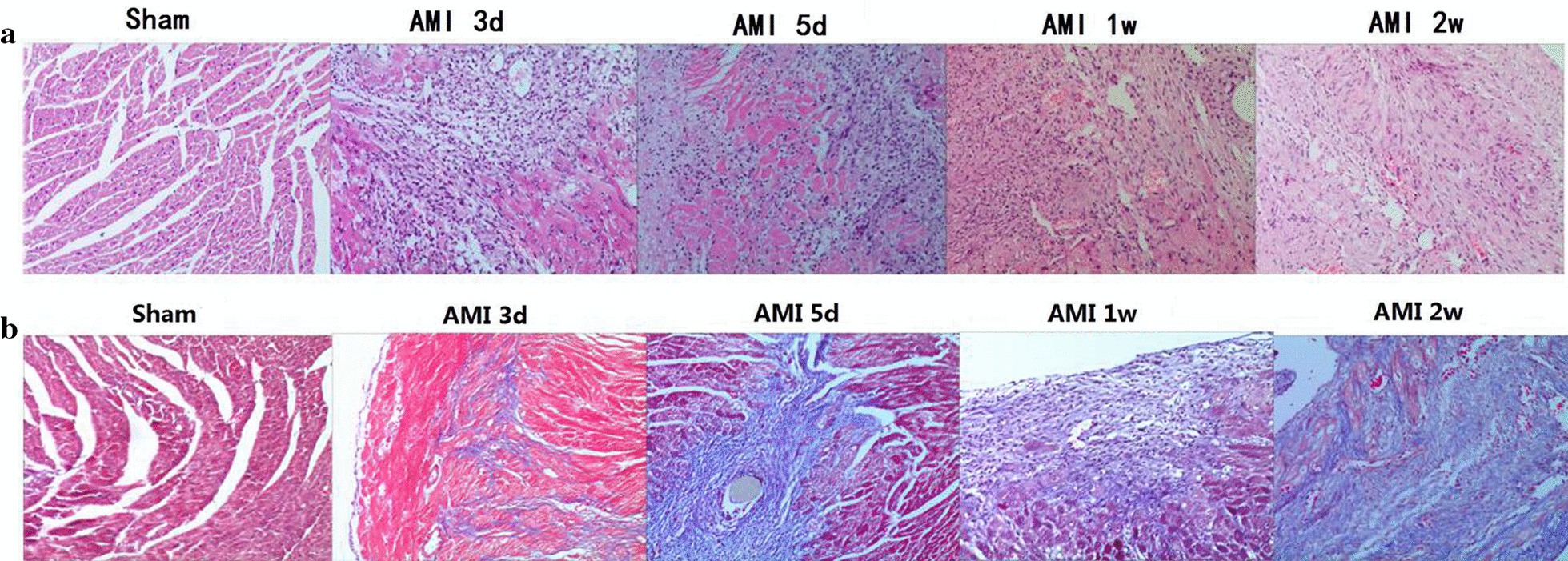
HE and Masson staining of myocardium AMI mice at different time points (× 200). a: HE staining, b: Masson staining. *AMI* acute myocardial infarction, *HE* hematoxylin–eosin staining

In the AMI group, myocardial cell necrosis and inflammatory cell infiltration occurred at 3 days. Inflammatory cell infiltration was the most obvious at 5 days, inflammatory cell infiltration decreased, and little fibrosis appeared at 1 week. A few inflammatory cells and obvious fibrosis appeared at 2 weeks. Masson staining indicated that myocardial collagen deposition increased with time and reached its peak at 2 weeks. No obvious abnormalities were found in the myocardial pathological analysis of the Sham group mice. The pathological changes are shown in Fig. 6.

### Effect of B cells on myocardial collagen metabolism after acute myocardial infarction in mice and its possible mechanism

#### Cytokine mRNA levels in myocardial tissue differed among the Sham group, WT group and BKO group

**Fig. 7:**
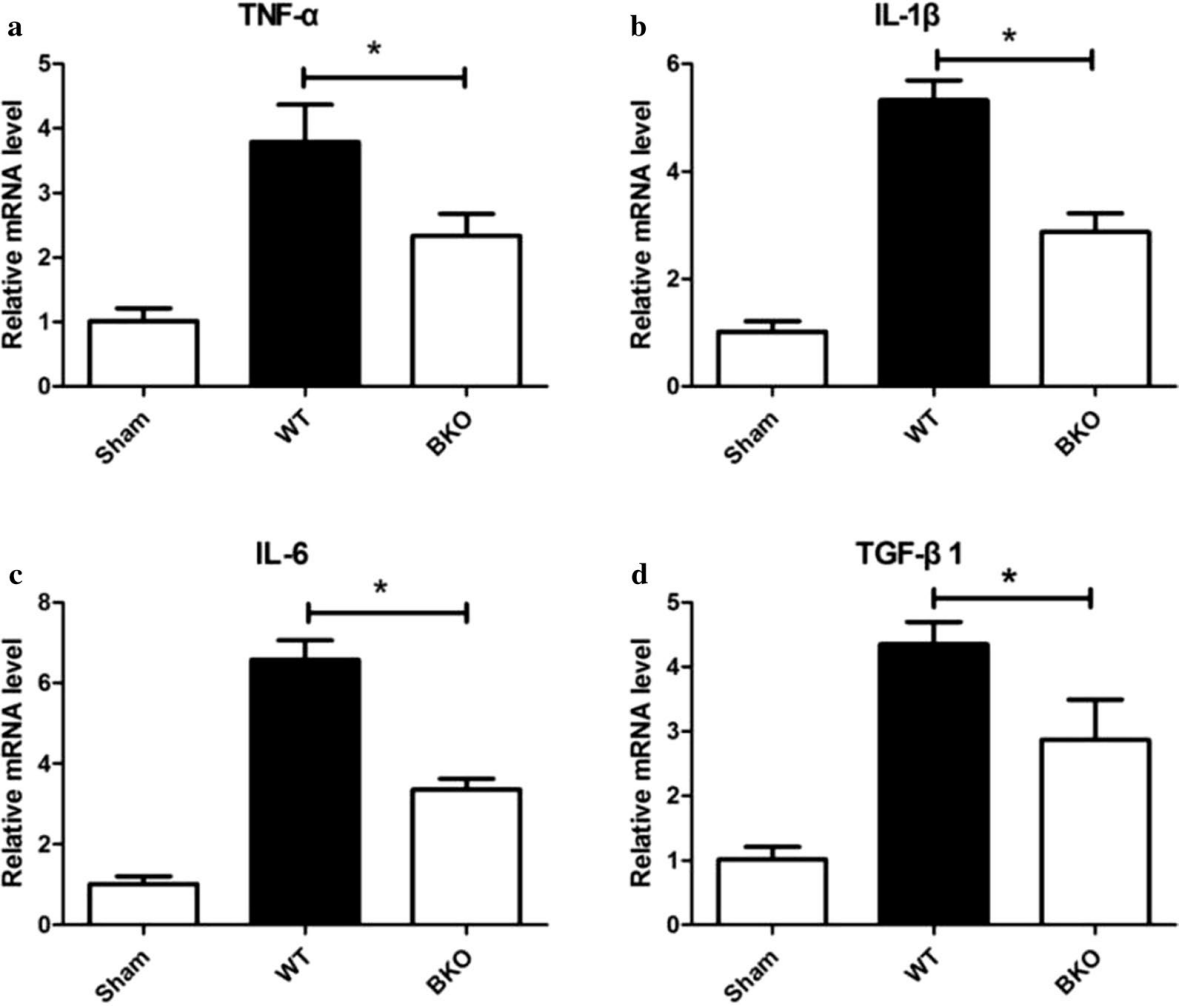
The relative expression level of cytokine mRNA in the myocardium of the Sham, WT, and BKO group. *P < 0.05, the expression level of cytokine mRNA of the Sham group was compared with WT and BKO groups. *WT* wild-type, *BKO* B-cell knockout, *TNF-α* tumour necrosis factor-α, *IL-1β* interleukin-1β, *IL-6* interleukin-6, *TGF-β1* transforming growth factor-β1

In the 5-day subgroup of AMI, the mRNA levels of TNF-α (2.33 ± 0.35VS3.79 ± 0.58), IL-1β (2.87 ± 0.35vs 5.32 ± 0.37), IL-6 (3.35 ± 0.28 vS6.58 ± 0.48), and TGF-β1 (2.87 ± 0.62 vS4.35 ± 0.35) in the BKO group were decreased compared with those in the WT group (*P* < 0.05) and were increased compared with those in the Sham group (*P* < 0.05) (Fig. 7).

#### Differences in peripheral blood cytokine concentrations in myocardial tissue among the Sham, WT and BKO groups

**Fig. 8:**
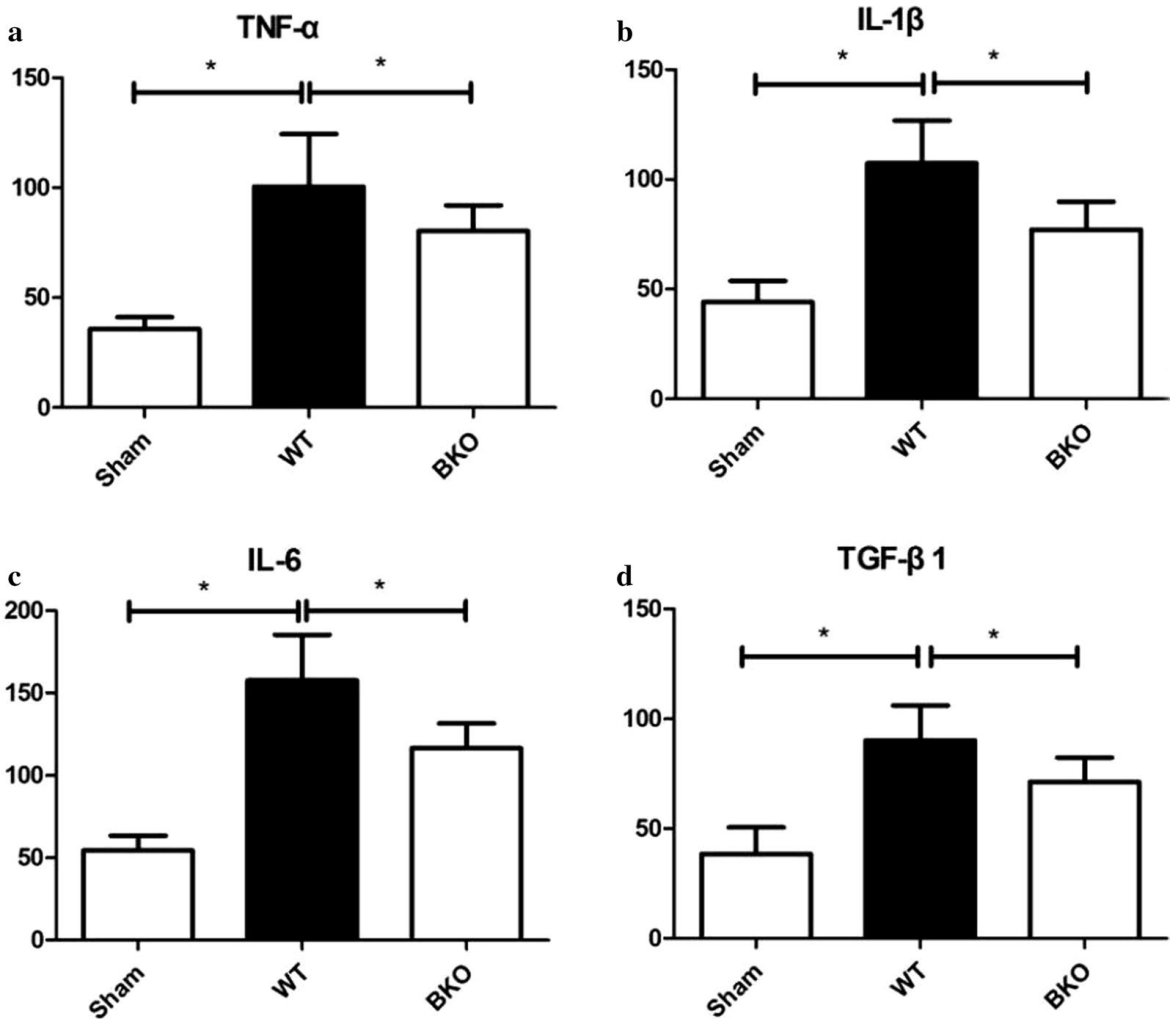
Peripheral blood cytokine concentrations in the myocardium of the Sham, WT, and BKO groups. *P < 0.05, the concentration of peripheral blood cytokine of the Sham group was compared with WT and BKO groups. *WT* wild-type, *BKO* B-cell knockout, *TNF-α* tumour necrosis factor-α, *IL-1β* interleukin-1β, *IL-6* interleukin-6, *TGF-β1* transforming growth factor-β1

In the 5-day subgroup of AMI, the concentrations of TNF-α, IL-1β, IL-6 and TGF-β1 in the peripheral blood of the BKO group were decreased compared with those in the WT group (all *P* < 0.05), which were increased compared with those in the Sham group (*P* < 0.05), as shown in Fig. 8.

#### mRNA levels of collagen metabolism in the myocardium

**Fig. 9:**
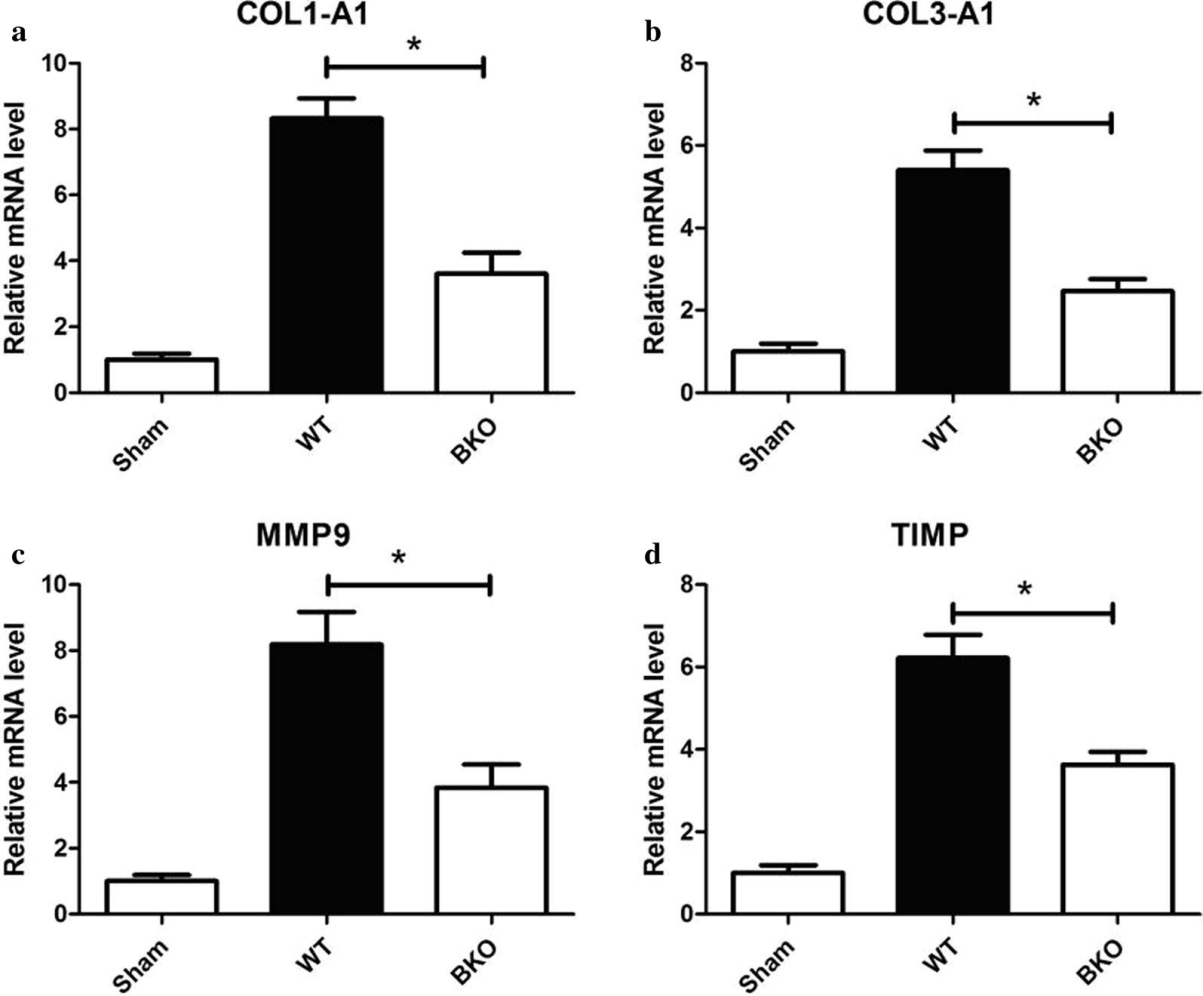
The relative expression levels of collagen metabolism index mRNA in the myocardium of AMI mice, *P < 0.05, the relative expression level of collagen metabolism index mRNA of the Sham group was compared with WT and BKO groups. *WT* wild-type, *BKO* B-cell knockout, *COL1-A1* collagen alpha-1(I), *COL3-A1*: collagen alpha-1(III), *MMP9* matrix metalloproteinase 9, *TIMP* tissue inhibitor of matrix metalloproteinase.

The mRNA levels of COL1-A1 (3.60 ± 0.65vs8.33 ± 0.60), COL3-A1 (5.40 ± 0.47vs2.46 ± 0.30), MMP9 (3.84 ± 0.71vs 8.19 ± 0.99) in the AMI group, and TIMP (3.63 ± 0.31vs6.23 ± 0.56) in the 2-week subgroup in the BKO group were decreased compared with those in the WT group (*P* < 0.05) but increased compared with those in the Sham group (*P* < 0.05) (Fig. 9).

#### Comparison of colour Doppler echocardiography in the 2-week subgroup of the Sham, WT and BKO groups

**Fig. 10:**
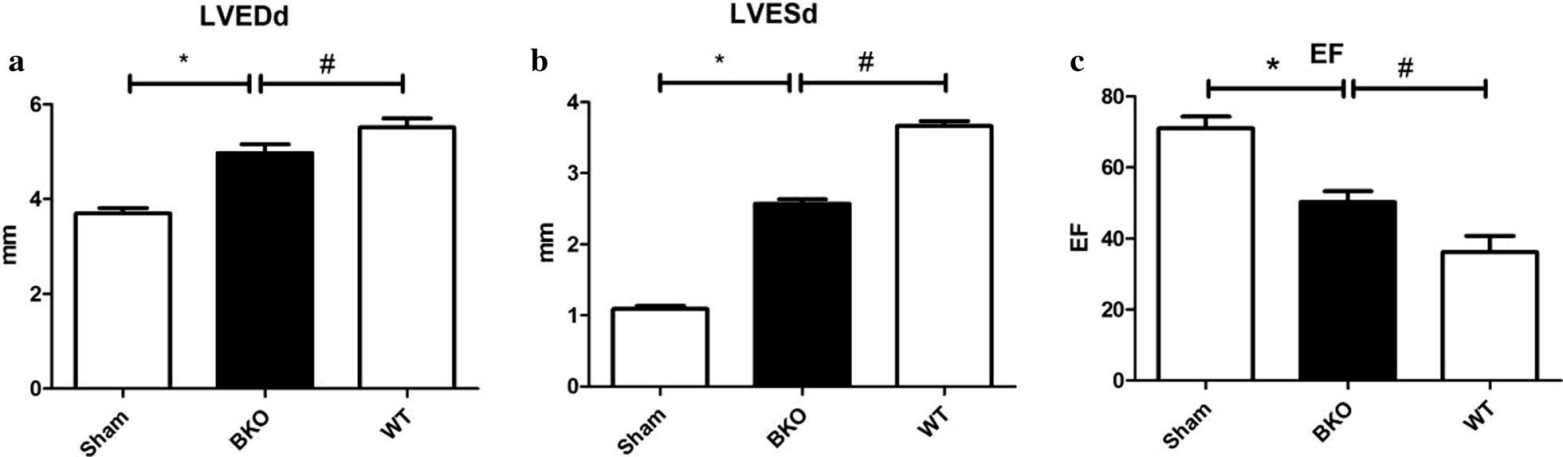
Color Doppler echocardiography in 2-week subgroup of Sham, WT, and BKO group. *P < 0.05, cardiac function indicators of BKO group was compared with Sham group, ^#^*P* < 0.05, cardiac function indicators of BKO group was compared with WT group. *WT* wild-type, *BKO* B-cell knockout, *LVEDd* left ventricular end-diastolic diameter, *LVESd* left ventricular end-diastolic diameter, *EF* ejection fraction

The left ventricular end-diastolic diameter (LVEDd) and left ventricular end-systolic diameter (LVESd) in the BKO group were smaller than those in the WT group (*P* < 0.05), and the EF value was higher than that in the WT group (*P* < 0.05). The LVEDd and LVESd were smaller than those in the Sham group, and the LVEF was lower than that in the Sham group. The difference was statistically significant (*P* < 0.05). The results are presented in Table 3 and Fig. 10.

**Table 3:** Color Doppler echocardiography in 2 weeks subgroup of Sham, WT, and BKO group

Indicators	Sham	BKO	WT
LVEDd (mm)	3.70 ± 0.12	4.97 ± 0.19*^#^	5.51 ± 0.19*
LVESd (mm)	1.09 ± 0.04	2.57 ± 0.06*^#^	3.67 ± 0.07*
EF (%)	71.00 ± 3.34	50.33 ± 3.01*^#^	36.17 ± 4.62*

*WT* wild-type, *BKO* B-cell knockout, *LVEDd* left ventricular end-diastolic diameter, *LVESd* left ventricular end-diastolic diameter, *EF* ejection fraction

^*^P < 0.05, cardiac function indicators of BKO group was compared with Sham group, #*P* < 0.05, cardiac function indicators of BKO group was compared with WT group. Data are represented by mean ± standard deviation

#### Comparison of pathological findings in the 2-week subgroup of the Sham group, WT group and BKO groups

**Fig. 11:**
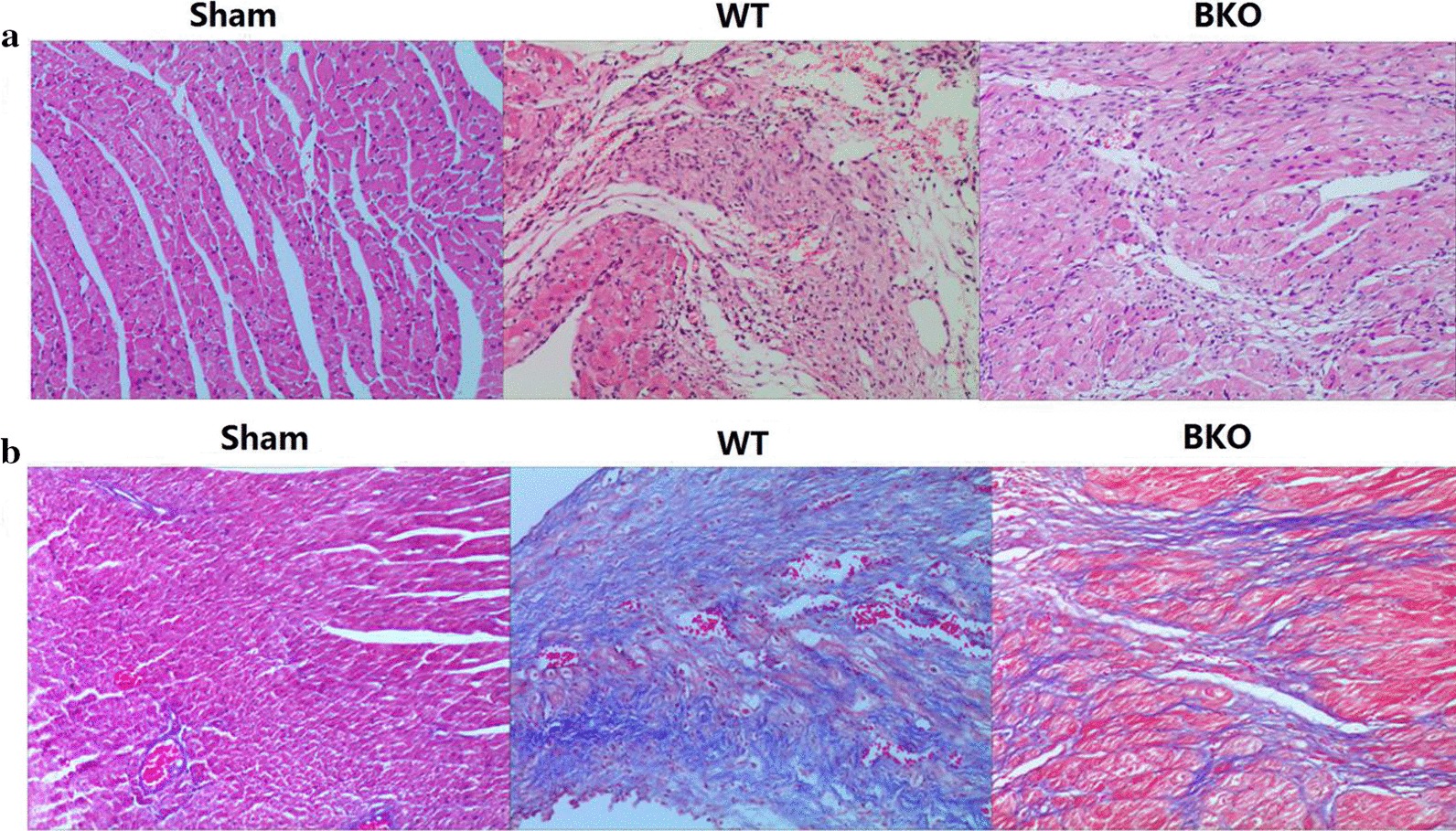
HE and Masson staining of the myocardium in 2-week subgroup of Sham, WT, and BKO groups (× 200). **a** HE staining, **b** Masson staining. *WT* wild-type, *BKO* B-cell knockout, *HE* hematoxylin–eosin staining

Compared with the myocardial pathology of the mice in the WT group at 2 weeks, the myocardial fibrosis in the BKO group was reduced, and the collagen content decreased. The fibrosis in the BKO and WT groups was more obvious than that in the Sham group. The results are shown in Fig. 11.

## Discussion

In recent years, the incidence of AMI has been on the rise, with very high mortality and disability rates, and has become one of the main causes of heart failure [26]. The primary pathogenesis of acute myocardial infarction (AMI) is unstable, ruptured coronary atherosclerotic plaques, thrombotic formation of platelet aggregation, sustained coronary artery occlusion and myocardial cell ischaemia/anoxic necrosis. Myocardial necrosis after AMI triggers the local inflammatory response and activates the immune system. Myocardial infarction not only directly leads to myocardial necrosis, but the secondary inflammatory immune response can also seriously damage cardiac function and can cause heart failure and various arrhythmias [4, 27–29].

As acquired immune cells, B cells secrete cytokines such as transforming growth factor-β1 (TGF-β1), tumour necrosis factor-α (TNF-α), interleukin-1β (IL-β1), and interleukin-6 (IL-6), which promote fibrosis progression. A large number of studies have shown that cytokines are involved in the inflammatory response and the fibrosis process of kidney, liver and lung tissues [30, 31]. In recent years, there have been reports on the involvement of B cells in the myocardial fibrosis process of cardiovascular diseases, but the mechanism of action of B cells and their cytokines in the myocardial infarction process after AMI is still unclear. Therefore, our study intended to establish a mouse AMI model by ligating the LAD artery of C57BL/6 mice to observe the changes in activated B cells and cytokines at different time points and the relationship between them. The study of an animal myocardial infarction model can help to explore its mechanism and treatment. Cardiovascular genes in mice are highly similar to those in humans [32], and current gene knockout and other gene technologies are achieved in mice. However, due to their small size, poor tolerance, and lack of convenience for surgery and operations, mouse myocardial infarction modelling has high requirements, often after a certain period of training to master the mouse AMI modelling technology. Ligation of the LAD artery is the mainstream method for the production of AMI models in mice [33], which requires a series of processes such as management of anaesthesia, endotracheal intubation, ventilator-assisted breathing, thoracotomy, pre-exposure of the descending branch, pre-ligation of the descending branch, chest closure, resuscitation, removal of the ventilator and so on.

In a study of patients with heart failure after myocardial infarction and various reasons, it was found that various anti-myocardial antibodies exist in myocardial tissue. Endothelial cells are damaged after myocardial ischaemia and hypoxia, secreting various cytokines, such as chemokines and inflammatory mediator factors. The ischaemic necrosis of the myocardium exposes new antigens, which trigger the immune response, antibody action and immune cell infiltration [34]. Moreover, the congenital immune system plays an important role in the myocardial fibrosis process. Neutrophils are first released due to damaged endothelial cells, and chemokines, growth factors and chemical signals affect the damaged myocardium. Then, natural killer cells, dendritic cells, mononuclear macrophages infiltrate into the damaged heart tissue, secreting cytokines and releasing oxygen free radicals, causing acute inflammation, and increasing infiltration of T cells and B cells into damaged areas. By secreting cytokines, producing antibodies and presenting antigens, B cells further aggravate myocardial injury under the combined action of inflammatory cytokines secreted by various activated immune cells [35–37]. In addition, B cells not only damage the myocardium but are also related to myocardial fibrosis after myocardial injury. In the B cell-deficient mouse cardiomyopathy model, it was found that with the decrease in TNF-α, serum collagen I and III levels decreased, and the amount of collagen fibres deposited in the extracellular matrix decreased [23]. Thus, it can be inferred that B cells have the role of promoting myocardial fibrosis. Zouggari et al. found that myocardial B cells expressed Ccl7, a chemokine recruited to myocardial infiltration by CCR2 receptor-mediated monocytes, which could lead to myocardial damage. After injection of anti-CD20 antibody, monocyte infiltration was reduced, and myocardial damage was alleviated [25]. However, Goodchild et al. reported that intramyocardial injection of bone marrow-derived B lymphocytes into SD rats with myocardial infarction is beneficial to cardiac function because it reduced in situ cell apoptosis and helped maintain the ejection fraction [38]. The two studies drew different conclusions about B cells because Goodchild et al. used immature B cells, while Zouggari et al. used anti-CD20 antibodies to exhaust mature B cells.

In our study, BKO mice were used to investigate the effect of B-cell deletion on myocardial collagen deposition after AMI. The results showed that B-cell deletion reduced the expression of the cytokines TNF-α, IL-1β, IL-6, and TGF-1β, decreased myocardial collagen synthesis after AMI, alleviated myocardial fibrosis, improved left ventricular remodelling, and maintained the left ventricular ejection fraction. The BKO mice used in this study completely lacked the entire B-cell system, and the B cells were removed from the source, which is different from the elimination of B cells by drug depletion, including anti-CD20, anti-CD22, anti-BAFF and other antigens that exhaust the blood circulation and express corresponding antigens. B-cell factors could be completely excluded.

## Conclusion

Activated B cells promote cytokine (TNF-α, IL-1β, IL-6, TGF-1β) secretion and may participate in the pathological process of myocardial injury after AMI, which is related to myocardial fibrosis after AMI. Moreover, cytokines affect myocardial collagen metabolism after AMI and promote myocardial I collagen and III expression, which seriously damage cardiac structure and ventricular diastolic and systolic function and eventually cause heart failure. However, further cell experiments as well as clinical experiments need to be conducted to verify our conclusions.

## Data Availability

The datasets used and/or analysed during the current study are available from the corresponding author on reasonable request.

## Abbreviations

AMI: Acute myocardial infarction
BAFF: B cell activating factor
BKO: B-cell knockout
COL1-A1: Collagen alpha-1(I)
COL3-A1: Collagen alpha-1(III)
ELISA: Enzyme-linked immunosorbent assay
HE: Hematoxylin–eosin staining
IL-1β: Interleukin-1β
IL-6: Interleukin-6
LAD: Left anterior descending
LSD: Least significant difference
LVEDd: Left ventricular end-diastolic diameter
LVESd: Left ventricular end-systolic diameter
MMP9: Matrix metalloproteinase 9
RT-PCR: Real-time fluorescence quantitative polymerase chain reaction
M ± SD: Mean ± standard deviation
TIMP-1: Tissue inhibitor of matrix metalloproteinase-1
TNF-α: Tumour necrosis factor-α
TGF-β1: Transforming growth factor-β1
WT: Wild-type
